# Characterization of the complete mitochondrial genome of *Chrysochir aureus* and phylogenetic studies of Sciaenidae

**DOI:** 10.1080/23802359.2020.1870900

**Published:** 2021-02-09

**Authors:** Xiaolong Yin, Xuepeng Li, Jian Chen, Kuo Tian, Hua Zhang, Pengxiang Yuan

**Affiliations:** a Marine Science and Technology College, Zhejiang Ocean University, Zhoushan, China; b Key Laboratory of Tropical Marine Bio-resources and Ecology, Chinese Academy of Sciences, Guangzhou, China; c Zhoushan Fisheries Research Institute of Zhejiang Province, Zhoushan, China; d College of Life Sciences, Yantai University, Yantai, China

**Keywords:** *Chrysochir aureus*, mitochondrial genome, phylogenetic analysis

## Abstract

The complete mitochondrial genome of *Chrysochir aureus* was sequenced. The full length of the mitochondrial genome was 16,501 bp, including 13 protein-coding genes (PCGs), two ribosomal RNAs, 22 transfer RNA genes, a non-coding control region (CR) and one origin of replication on the light-strand (OL). The total nucleotide composition of mitochondrial DNA was 26.95% A, 29.99% C, 26.29% T, and 16.77% G. Twelve PCGs used the canonical ATG as their initiation codon, whereas COI gene started with an alternative start codon GTG. The mitochondrial genome of *C. aureus* described in this study could be a useful basis for management of this species and laid a foundation for further research involved with phylogenetic relationship within Sciaenidae.

*Chrysochir aureus* is widely distributed in southeast India and Sri Lanka to southern China (Yong et al. 2010), it is an important economic fish in many coastal cities and is marketed fresh as well as dried salted (Bianchi 1984). Due to its illegible appearance and high price, fishermen usually use some miscellaneous fish to make up the quantity unintentionally or intentionally; besides, phylogenetic position of *C. aureus* has always been controversial (Yong et al. 2010; Wang et al. 2017). In this study, we described the complete mitochondrial genome of *C. aureus* and explored its phylogenetic position within Sciaenidae, to gain its molecular information which was expected to contribute to purchasing management as well as further phylogenetic studies on its related species.

An individual specimen of *C. aureus* was sampled by commercial bottom trawling in the South China Sea (N28°00′30.56″, E121°48′79.59″) and stored in the Research Center of Zhejiang Ocean University with accession number 20190319YXT78. The total genomic DNA was extracted from a portion of the epaxial musculature using the phenol–chloroform method (Barnett and Larson 2012). The complete mitogenome of *C. aureus* was amplified with the help of universal primers for marine fish species (Cheng et al. 2012; Shao et al. 2014). Fragments generated from PCR amplification were sequenced using Sanger sequencing technology. Sequenced fragments were assembled to create the complete mitogenome using CodonCode Aligner 5.1.5 (CodonCode Corporation, Dedham, MA). The complete mitogenome was annotated using the software of Sequin (version 15.10, http://www.ncbi.nlm.nih.gov/Sequin/). Transfer RNA (tRNA) genes and their potential cloverleaf structures were identified using tRNAscan-SE 1.21 (Lowe and Eddy 1997). 

The complete mitogenome of *C. aureus* was 16,501 bp in length (GenBank accession number MW026682), containing 13 protein-coding genes (PCGs), two ribosomal RNA genes (12S and 16S), 22 tRNA genes, one origin of replication on the light-strand (OL) and a putative control region (CR). The overall base composition was 26.95% A, 29.99% C, 26.29% T, and 16.77% G, respectively, with a slight AT bias of 52.24%. The gene arrangement, composition, and size were quite similar to the teleost fish mitogenomes published previously (Jing et al. 2010; Zhu et al. 2018). The total length of 13 PCGs of *C. aureus* mitogenome was 11,409 bp, encoding 3792 amino acids. Similar to the typical vertebrate mitogenome (Miya and Nishida 2000), 12 of them were located on the H-strand, except for *ND6* which was detected on the L-strand. All of the PCGs used the canonical ATG initiation codon with the exception of *COI* gene, which started with an alternative start codon GTG. Seven PCGs (*ND1*, *ND2*, *ATP8*, *ATP6*, *COIII*, *ND4L*, and *ND6*) terminated with the stop codon TAA, two (*COI*, *ND5*) with AGA, one (*ND3*) with TAG, the genes ended with a single T were *COII*, *ND4*, and *Cytb*, the presence of an incomplete stop codon is a common phenomenon in vertebrate mitochondrial genes (Zhu et al. 2018; Lee et al. 2019). Twenty-two tRNAs that dispersed between rRNAs and PCGs were identified by their specific anticodon sequences. All the tRNAs were capable of folding into the archetypal cloverleaf structure except for tRNA^Ser (AGC)^ that lacked a dihydrouridine arm (Garey and Wolstenholme 1989). The lengths of the two rRNA genes were 954 bp (12SrRNA) and 1707 bp (16SrRNA), respectively, which located between the tRNA^Phe^ and tRNA^Leu(UUA)^ and interposed by the tRNA^Val^. The CR was detected between tRNA^phe^ and tRNA^Pro^, consisting of 821 nucleotides, G-C content (35.45%) was significantly lower than that of A-T (64.55%).

Although the complete mitogenome had been reported by Wang et al. (2017) that revealed *C. aureus* was closely related to the species of genera *Nibea* instead of *Protonibea*, phylogenetic relationship of *C. aureus* remains uncertain and related research still needs further development. In this study, a total of 29 mitogenomes were downloaded from NCBI website for constructing by far the most comprehensive phylogenetic tree of Sciaenidae based on 13 PCGs. The ML tree showed different clustering results and indicated *C. aureus* was first clustered with *Protonibea diacanthus* (Figure 1), being consistent with previous studies (Chen 2007; Yong et al. 2010). The present study might give more thought to phylogenetic position of *C. aureus* and was expected be helpful for further phylogenetic analysis of Sciaenidae.

**Figure 1.**
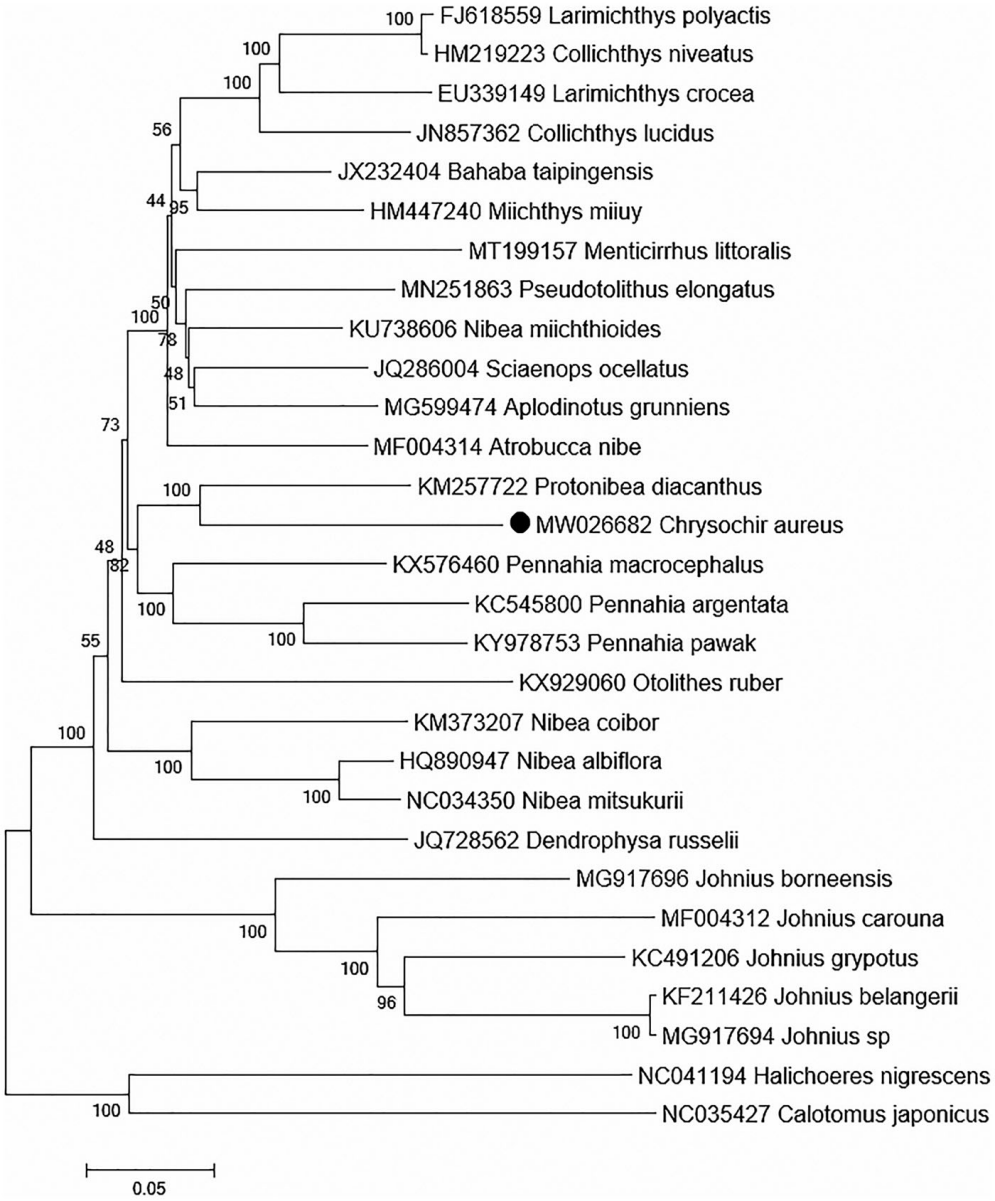
Maximum-likelihood (ML) tree of 27 Sciaenidae species based on 13 PCGs, two Labridae species are selected as outgroup. The bootstrap values are based on 1000 resamplings. The number at each node is the bootstrap probability. The number before the species name is the GenBank accession number. The genome sequence in this study is labeled with a black dot.

## Data Availability

The genome sequence data that support the findings of this study are openly available in GenBank of NCBI at https://www.ncbi.nlm.nih.gov/ under the accession no. MW026682.

## References

[CIT0001] Barnett R, Larson G. 2012. A phenol–chloroform protocol for extracting DNA from ancient samples. Methods Mol Biol. 840:13–19. 22237516 10.1007/978-1-61779-516-9_2

[CIT0002] Bianchi G. 1984. FAO species identification sheets for fishery purposes, Western Indian Ocean (fishing area 51)/edited by W. Fischer and G. Bianchi. Eur J Radiol. 45:208–213.

[CIT0003] Chen Q. 2007. Molecular phylogeny of the Sciaenidae in China. Guangzhou: Jinan University.

[CIT0004] Cheng YZ, Xu TJ, Jin XX, Tang D, Wei T, Sun YY, Meng FQ, Shi G, Wang RX. 2012. Universal primers for amplification of the complete mitochondrial control region in marine fish species. Mol Biol. 46(5):727–730. 23156681

[CIT0005] Garey JR, Wolstenholme DR. 1989. Platyhelminth mitochondrial DNA: evidence for early evolutionary origin of a tRNA (serAGN) that contains a dihydrouridine arm replacement loop, and of serine-specifying AGA and AGG codons. J Mol Evol. 28(5):374–387. 2545889 10.1007/BF02603072

[CIT0006] Jing H, Zhang D, Hao J, Huang D, Cameron S, Zhu C. 2010. The complete mitochondrial genome of the yellow coaster, *Acraea issoria* (Lepidoptera: Nymphalidae: Heliconiinae: Acraeini): sequence, gene organization and a unique tRNA translocation event. Mol Biol Rep. 37(7):3431–3438. 20091125 10.1007/s11033-009-9934-3

[CIT0007] Lee S-H, Kang C-B, Shin M-H, Lee S-H, Yoon M, Kim HJ. 2019. The complete mitochondrial genome of a marbled eelpout *Lycodes raridens* (Perciformes: Zoarcidae). Mitochondrial DNA Part B. 4(2):4043–4044. 33366309 10.1080/23802359.2019.1688698 PMC7707741

[CIT0008] Lowe T, Eddy S. 1997. tRNAscan-SE: a program for improved detection of transfer RNA genes in genomic sequence. Nucleic Acids Res. 25(5):955–964. 9023104 10.1093/nar/25.5.955 PMC146525

[CIT0009] Miya M, Nishida M. 2000. Use of mitogenomic information in teleostean molecular phylogenetics: a tree-based exploration under the maximum-parsimony optimality criterion. Mol Phylogenet Evol. 17(3):437–455. 11133198 10.1006/mpev.2000.0839

[CIT0010] Shao W, Wei L, Li L, Wang H, Lin Z, Chen J. 2014. Universal DNA primers for amplification complete mitochondrial genome for sturgeons. Conserv Genet Resour. 6(2):305–307.

[CIT0011] Wang Y, Chai X, Hu Z, Xu T. 2017. Complete mitochondrial genome of the golden drum *Chrysochir aurenus* (Perciformes, Sciaenidae) and its phylogeny. Mitochondrial DNA Part A. 28(1):54–55. 10.3109/19401736.2015.1110795 26680686

[CIT0012] Yong Z, Chun-Yan MA, Ling-Bo MA, Yong NI, Ang-Lv S. 2010. Molecular phylogenetic relationships of 13 Sciaenidae species in China sea areas based on 16SrRNA fragment sequences. Mar Fish. 32:276–282.

[CIT0013] Zhu K, Lü Z, Liu L, Gong L, Liu B. 2018. The complete mitochondrial genome of *Trachidermus fasciatus* (Scorpaeniformes: Cottidae) and phylogenetic studies of Cottidae. Mitochondrial DNA Part B. 3(1):301–302. 33474152 10.1080/23802359.2018.1445480 PMC7799978

